# Kindlin-2 could influence breast nodule elasticity and improve lymph node metastasis in invasive breast cancer

**DOI:** 10.1038/s41598-017-07075-1

**Published:** 2017-07-28

**Authors:** Xiaowei Xue, Junlai Li, Wenbo Wan, Xianquan Shi, Yiqiong Zheng

**Affiliations:** 1 Department of Ultrasound, The Southern Building, Chinese PLA General Hospital, Beijing, 100853 China; 2 Department of Ultrasound, Chinese PLA General Hospital, Beijing, 100853 China; 3 Department of Ultrasound, Beijing Friendship Hospital, Beijing, 100050 China; 4 Department of General Surgery, Chinese PLA General Hospital, Beijing, 100853 China

## Abstract

This study investigated the relationship between quantitative parameters of shear wave elastography (SWE, maximum elasticity [Emax], minimum elasticity [Emin], mean elasticity [Emean]), collagen intensity and Kindlin-2 expression in benign and malignant breast nodules, and if Kindlin-2 expression is related with lymph node metastasis. A total of 102 breast nodules from 102 patients were included in our study who underwent ultrasound elastography before surgery or core needle biopsy. There was a significant difference between benign and malignant breast nodules in Emax, Emean, collagen intensity and Kindlin-2 expression, but it had no difference in Emin. Collagen intensity and Kindlin-2 expression both correlated positively with Emax, but not with Emean. Among 38 malignant breast nodules, the average Emax of the metastasis group was higher than that of the non-metastasis group, but it had no statistical significance. Compared with the non-metastasis group, Kindlin-2 expression was considerably higher in the metastasis group. However, there was no difference in collagen intensity between the metastasis group and the non-metastasis group. In conclusion, Kindlin-2 and collagen might contribute to breast nodule elasticity through molecular mechanisms. In breast cancer, overexpression of Kindlin-2 might be a risk factor for lymph node metastasis.

## Introduction

Stiffness is a key characteristic of tissues and organs, which has historically served as the basis for clinical examinations, such as palpation, especially in breast abnormalities. As clinicians looked for ways to more systematically define stiffness, ultrasound elastography was developed. In breast, elastography has been applied in focal diseases and a breast nodule that is firm and hard is associated with an increasing risk of malignancy^1^. It had first been introduced to evaluate tissue stiffness in 1997^2^. Elastography has been gradually accepted by clinicians^3–5^. However, conventional elastography suffers from lack of reproducibility, significant inter-operator variability, and yields subjective, semi-quantitative measurements. To overcome these limitations, SWE has been developed, which provides reproducible, quantitative data. This new method of obtaining elastographic images is based on the combination of a radiation force induced in a tissue by an ultrasonic beam and an ultrafast imaging sequence capable of catching in real time the propagation of the resulting shear wave^6^. SWE yields quantitative parameters Emax, Emin and Emean, which are measured in units of kilopascal (kPa) and reproducible information on solid breast lesions.

The stiffness of lesions is closely associated with collagen content, and proteins which can promote collagen expression and tissue fibrosis^7^. Collagen is an important constituent of the extracellular matrix (ECM) and contributes to breast cancer formation, invasion, and metastasis^8–10^. Kindlins are a group of FERM domain-containing proteins that have recently gained attention for their ability to bind and activate integrins. Kindlin-2, a member of Kindlins protein family, is evolutionarily conserved and widely expressed as an important regulator of integrin mediated ECM interaction^11–13^. Kindlin-2 is also reported to be an important factor in the regulation of podocyte-matrix adhesion, and matrix deposition of fibronectin and collagen type I^14^. Other studies found that Kindlin-2 could activate the TGF-β/Smad signaling, contributing to the pathogenesis of pancreatic ductal adenocarcinoma^15^ and tubulointerstitial fibrosis by stimulating the production of collagen^16^.

The incidence of breast cancer is on the rise in China, which affects women’s the health and life quality^17, 18^, and accurate, timely diagnosis is very important for millions of patients. The latest development in breast elastography has made SWE available, which offers higher diagnostic sensitivity and specificity, compared to conventional ultrasound. The aims of present study were to investigate if SWE can be used to determine the degree of fibrosis of breast nodules and if Kindlin-2 expression is correlated with the degree of fibrosis as well as SWE results. We also wanted to determine if nodule stiffness and Kindlin-2 expression can be used as indicators of lymph node metastasis in breast cancer.

## Results

### SWE diagnosis of patients with breast nodules

**Figure 1:**
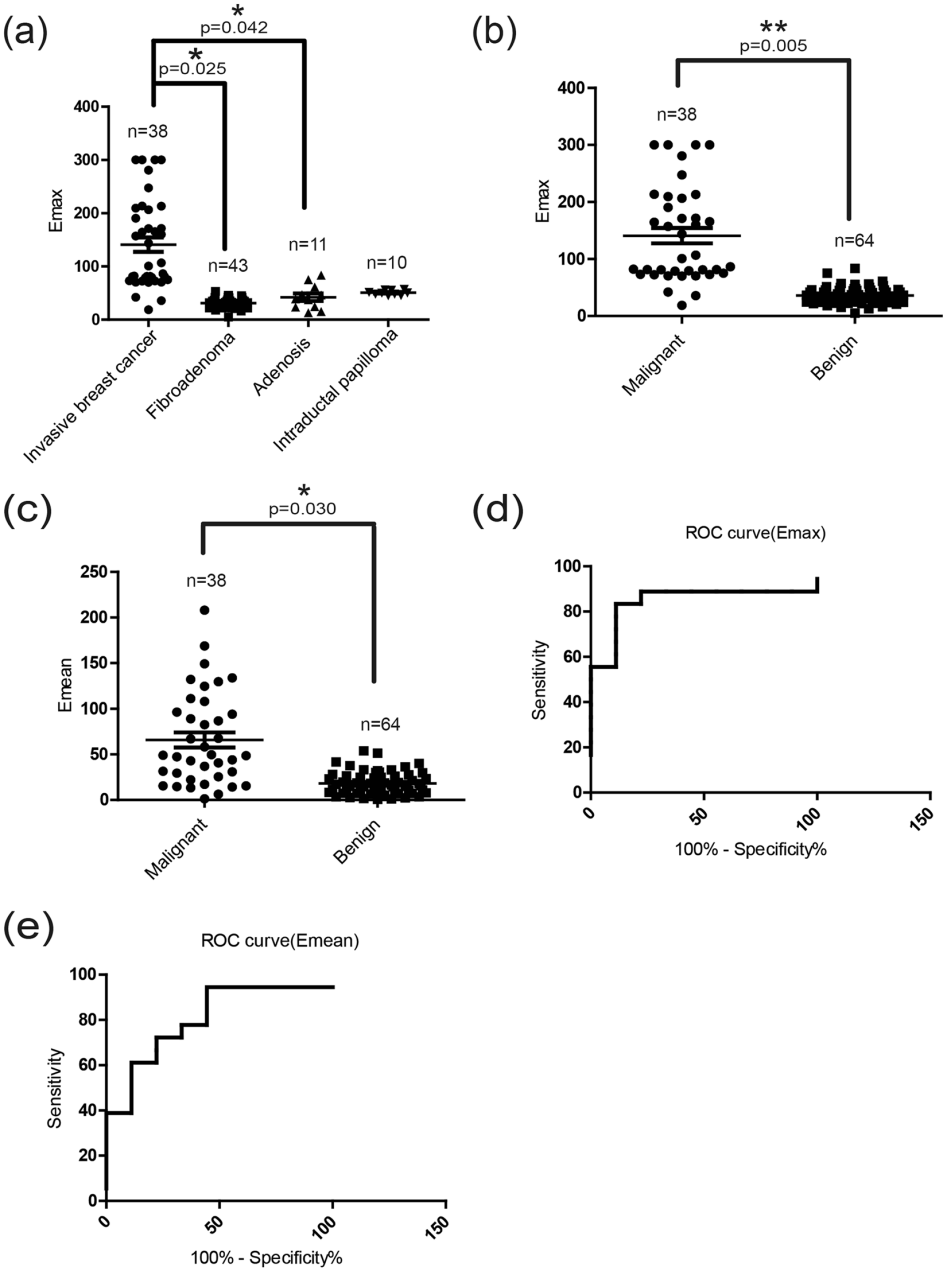
SWE diagnosis of patients with breast nodules. (**a**) Emax of different pathologies (invasive breast cancer, fibroadenoma, adenosis and intraductal papilloma). The Emax of invasive breast cancer was higher than that of fibroadenomas, adenoses and intraductal papillomas, but it had no statistical significance with intraductal papillomas (p = 0.100). (**b**) The Emax of malignant breast nodules was significantly higher than that of benign breast nodules. (**c**) The Emean of malignant breast nodules was significantly higher than that of benign breast nodules. (**d**) ROC curve for Emax in diagnosing benign and malignant lesions. (**e**) ROC curve for Emean in diagnosing benign and malignant lesions. Values are means ± SD. Comparisons between two groups were made using Student’s *t*-test. Differences between more than two groups were compared using one-way ANOVA. *p < 0.05, **p < 0.01. Emax, Maximum elasticity; Emean, Mean elasticity; ROC, Receiver operating characteristic; SD, Standard deviation; SWE, shear wave elastography; ANOVA, analysis of variance.

Table 1 shows the SWE measurements of breast lesions with different pathologies. The Emax of invasive breast cancer was significantly higher than that of fibroadenomas (p = 0.025) and adenoses (p = 0.042). Although the Emax of invasive breast cancer was also higher than that of intraductal papillomas, the difference was not statistically significant (p = 0.100) (Fig. 1(a)). There was no significant difference in Emin or Emean values between the four pathologies. The Emax, Emin and Emean values of malignant and benign lesions can be seen in Table 2. As Fig. 1 shows, malignant breast nodules had higher Emax (139.74 ± 98.77 kPa vs. 35.42 ± 19.42 kPa, p = 0.005) (Fig. 1(b)) and Emean (65.91 ± 60.71 kPa vs. 18.29 ± 13.83 kPa, p = 0.030) (Fig. 1(c)) than benign nodules. The optimal cutoff values of Emax and Emean for the highest Youden index (also known as the correct diagnostic index, it defines the ability to differentiate the pathologically confirmed breast cancer from suspected breast cancer) were 55.86 kPa and 20.40 kPa for predicting malignant breast nodules, which respectively yielded 83.30% and 72.20% sensitivity (the percentage of patients with suspected breast cancer in patients with pathologically confirmed breast cancer), 88.90% and 77.80% specificity (the percentage of patients with suspected benign disease in patients with pathologically confirmed benign disease), 88.20% and 76.50% positive predictive value (the percentage of patients with pathologically confirmed breast cancer in patients with suspected breast cancer), 84.19% and 73.67% negative predictive value (the percentage of patients with pathologically confirmed breast cancer in patients with suspected breast cancer), and 86.10% and 75.00% accuracy (the percentage of patients with pathologically confirmed breast cancer and breast benign disease in all studied cases). The area under the curves for Emax and Emean was 0.85 (Fig. 1(d)) and 0.80 (Fig. 1(e)), respectively.

**Table 1:** Emax, Emin and Emean of different pathology breast nodules.

Pathological diagnosis	Emax (kPa)	Emin (kPa)	Emean (kPa)
Invasive breast cancer (n = 38)	139.74 ± 98.77	20.20 ± 39.87	65.91 ± 60.71
Fibroadenoma (n = 43)	31.28 ± 9.12	5.83 ± 3.98	14.15 ± 4.25
Adenosis (n = 11)	42.22 ± 28.29	14.21 ± 11.79	24.55 ± 19.83
Intraductal papilloma (n = 10)	50.77 ± 5.03	7.04 ± 1.00	20.94 ± 2.52

**Table 2:** Emax, Emin and Emean of malignant and benign breast nodules.

	Emax (kPa)	Emin (kPa)	Emean (kPa)
Malignant	139.74 ± 98.77	20.20 ± 39.87	65.91 ± 60.71
Benign	35.42 ± 19.42	9.37 ± 8.91	18.29 ± 13.83
p Value	0.005**	0.285	0.030*

There were significant differences between malignant and benign breast nodules, *P < 0.05, **P < 0.01.

### SWE images and collagen and Kindlin-2 expression levels in malignant and benign breast nodules

**Figure 2:**
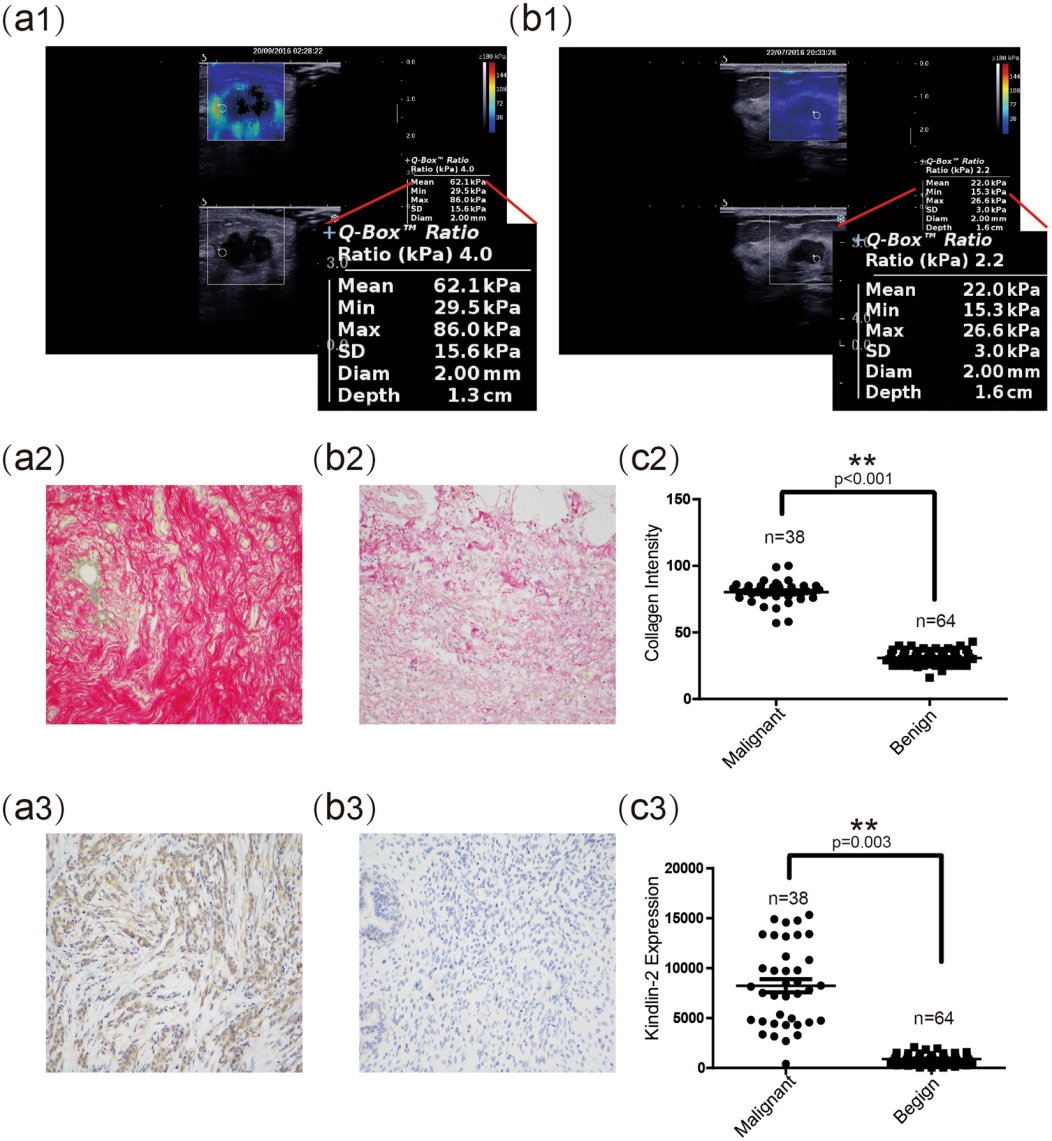
Evaluation of malignant and benign breast nodules by SWE and by histopathological staining for collagen and Kindlin-2 expression. (**a1**) (**b1**) Representative SWE images of malignant and benign breast nodules. (**a2**) (**b2**) Representative images from Sirius red staining (×200) show expression of collagen in malignant and benign breast nodules, respectively. (**c2**) Quantitative analysis shows that the average intensity of collagen in malignant breast nodules was significantly higher than that in benign breast nodules. (**a3**) (**b3**) Representative images of immunohistochemical staining (×200) show expression of Kindlin-2 in malignant and benign breast nodules, respectively. (**c3**) Quantitative analysis shows that the average expression (by integrated optical density of positive reactions) of Kindlin-2 in malignant breast nodules was significantly higher than that in benign breast nodules. Values are means ± SD. Comparisons between two groups were made using Student’s *t*-test. *p < 0.05, **p < 0.01. SWE, shear wave elastography; SD, standard deviation.

Representative SWE images of malignant and benign breast nodules are presented in Fig. 2(a1) and (b1), respectively. Malignant breast nodules were harder than benign breast nodules. Collagen intensity of malignant nodules was higher than that of benign nodules (80.25 ± 8.51 vs. 30.77 ± 5.32, p < 0.001) (Fig. 2(a2–c2)). Kindlin-2 expression of malignant nodules was also higher than that of benign nodules (8231.77 ± 4596.01 vs. 885.75 ± 417.77, p = 0.003) (Fig. 2(a3–c3)).

### Correlation of Emax and collagen intensity/Kindlin-2 expression in breast nodules

**Figure 3:**
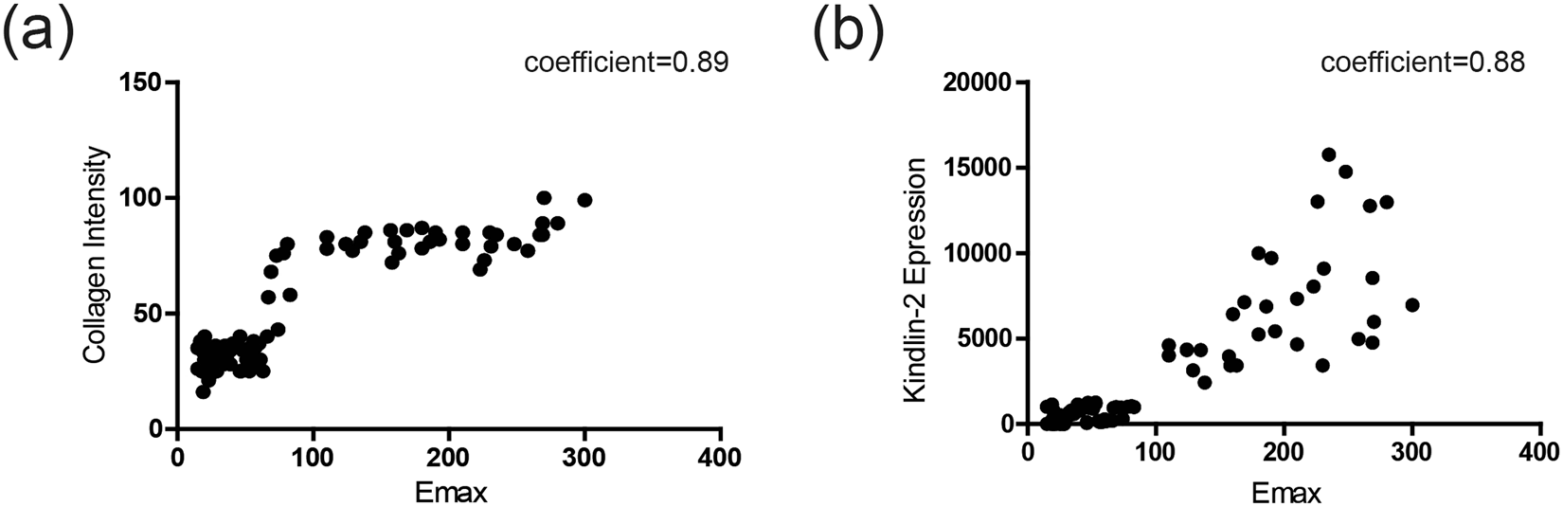
Correlation of Emax and collagen/Kindlin-2 expression in breast nodules. (**a**) Collagen intensity correlated positively with Emax (Coefficients = 0.89). (**b**) Kindlin-2 expression correlated positively with Emax (Coefficients = 0.88). Correlations were evaluated with Pearson’s test, and correlation coefficients were calculated. Coefficients ≥0.7 indicate a strong correlation; values ≥ 0.4 but < 0.7 indicate a moderate correlation; and values < 0.4 indicate a weak correlation. Emax, maximum elasticity.

Collagen intensity (Fig. 3(a)) and Kindlin-2 expression (Fig. 3(b)) correlated positively with Emax in all cases studied (coefficient = 0.89, p < 0.001 for collagen, and coefficient = 0.88 for Kindlin-2, p < 0.001) (Table 3).

**Table 3:** Correlation of collagen intensity and Kindlin-2 expression with Emax of breast nodules.

	Correlation coefficient	
	Collagen intensity	Kindlin-2 expression
Emax (kPa)	0.89	0.88
p Value	<0.001	<0.001

### Expression levels of collagen and Kindlin-2 in malignant breast nodules with or without lymph nodes metastasis

**Figure 4:**
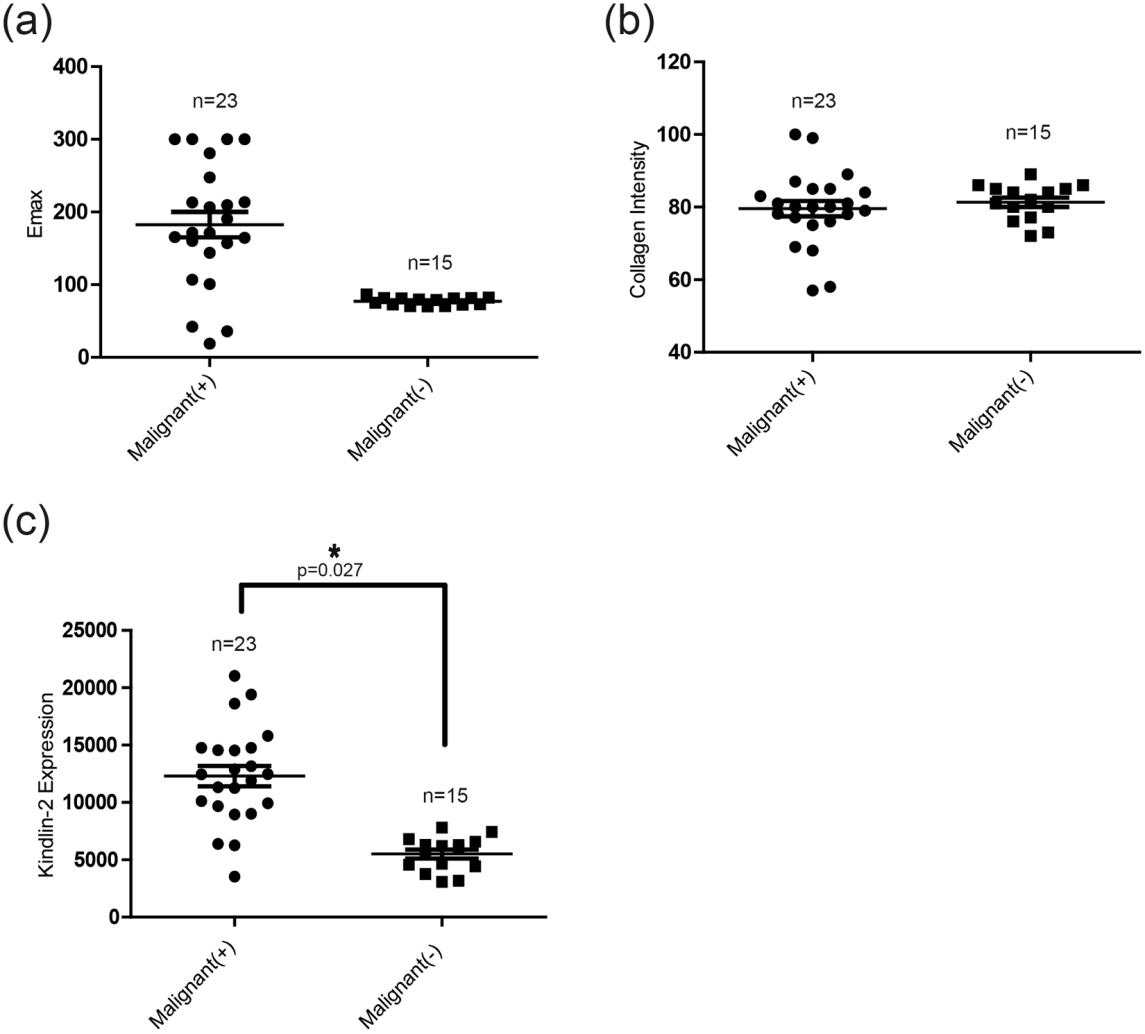
Expression levels of collagen and Kindlin-2 in malignant breast nodules with or without lymph nodes metastasis. (**a**) Emax of malignant breast nodules with lymph node metastasis were higher than that of nodules without lymph node metastasis, but it had no statistical significance (p = 0.110). (**b**) There was no difference in collagen intensity between the metastasis group and the non-metastasis group (79.54 ± 10.22 vs. 81.35 ± 5.10, p = 0.529). (**c**) Kindlin-2 expression of malignant breast nodules with lymph node metastasis was significantly higher than that of nodules without lymph node metastasis. Values are means ± SD. Comparisons between two groups were made using Student’s *t*-test. *p < 0.05, **p < 0.01. Emax, maximum elasticity; SD, Standard deviation.

In all 38 malignant nodules, the average Emax was higher in the metastasis group than in the non-metastasis group (182.66 ± 89.22 kPa vs. 77.11 ± 5.84 kPa, respectively), but it had no statistical significance (p = 0.110) (Fig. 4(a)). In all 38 malignant nodules, There was no difference in collagen intensity between the metastasis group and the non-metastasis group (79.54 ± 10.22 vs. 81.35 ± 5.10, p = 0.529) (Fig. 4(b)). However, Kindlin-2 expression was significantly higher in the metastasis group than in the non-metastasis group (12294.57 ± 2998.21 vs. 5502.31 ± 1669.94, p = 0.027) (Fig. 4(c)).

### Expression levels of p-FAK and p-Smad2 in malignant nodules with or without lymph nodes metastasis and in benign nodules

**Figure 5:**
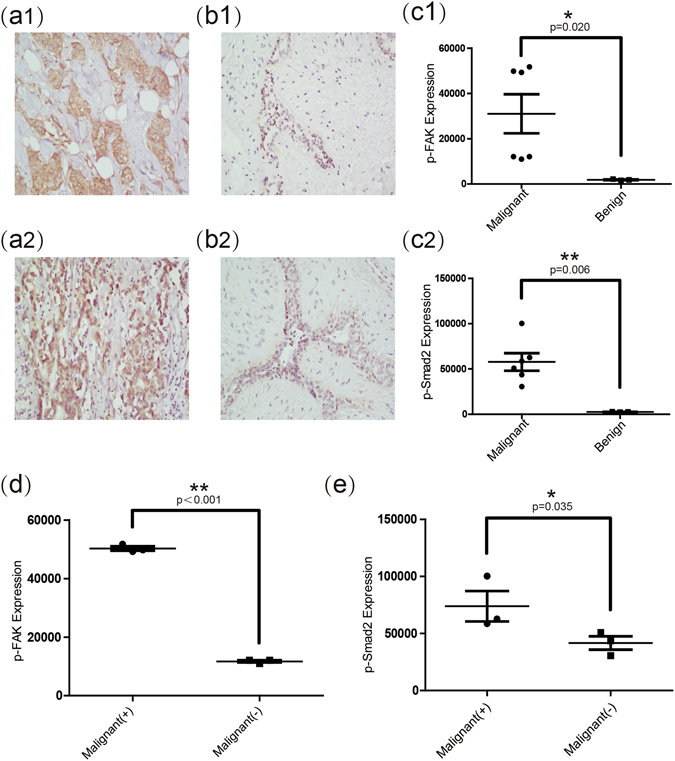
Expression levels of p-FAK and p-Smad2 in malignant nodules with or without lymph nodes metastasis, and in benign nodules. (**a1**) (**b1**) Representative images of immunohistochemical staining (×200) show expression of p-FAK in malignant and benign breast nodules. (**c1**) Quantitative analysis shows that the average expression (by integrated optical density of positive reactions) of p-FAK in malignant breast nodules was significantly higher than that in benign breast nodules. (**a2**) (**b2**) Representative images of immunohistochemical staining (×200) show expression of p-Smad2 in malignant and benign breast nodules. (**c2**) Quantitative analysis shows that the average expression (by integrated optical density of positive reactions) of p-Smad2 in malignant breast nodules was significantly higher than that in benign breast nodules. (**d**) The p-FAK expression of malignant breast nodules with lymph node metastasis was significantly higher than that without lymph node metastasis. (**e**) The p-Smad2 expression of malignant breast nodules with lymph node metastasis was significantly higher than that without lymph node metastasis. Values are means ± SD. Comparisons between two groups were made using Student’s *t*-test. *p < 0.05, **p < 0.01. SD, Standard deviation.

Given the differences that exist in the expression levels of Kindlin-2 in malignant and benign nodules, we investigated whether Kindlin-2 activates integrin and/or TGFβ signaling to enhance breast cancer elasticity and metastasis. To this end, we preliminarily examined the expression of p-FAK and p-Smad2 in nine cases (including 3 cases of benign nodules, 3 cases of malignant disease with metastasis, 3 cases of malignant disease without metastasis). Interestingly, the expression of p-FAK was higher in malignant nodules than in benign nodules (1847.14 ± 287.91 vs. 30986.63 ± 21150.24, p = 0.020) (Fig. 5(a1–c1)). The expression of p-Smad2 was also higher in malignant nodules than in benign nodules (2574.87 ± 225.66 vs. 57786.39 ± 23771.11, p = 0.006) (Fig. 5(a2–c2)). Compared with malignant non-metastasis group, the expression of both p-FAK and p-Smad2 were higher in metastasis group (50275.91 ± 1303.44 vs. 11697.36 ± 635.89, p < 0.001 for p-FAK expression (Fig. 5(d)), and 73890.35 ± 23007.70 vs. 41682.42 ± 10261.39, p = 0.035 for p-Smad2 expression (Fig. 5(e))). Taken together, these results suggested that Kindlin-2 might influence breast cancer elasticity and improve metastasis by activating integrin and TGFβ signaling pathways.

## Discussion

Elastography is a medical imaging modality that measures tissue stiffness, and is being used as an adjunct to conventional ultrasound. This technique facilitates the differentiation between benign and malignant lesions and helps refine the Breast Imaging-Reporting and Data System score (BI-RADS)^19–21^. A series of studies on the impact of SWE on the classification of breast lesions have shown its value in reclassifying BI-RADS 3 and 4a^22–24^. Emax is a SWE quantitative parameter that can inform clinicians about the stiffness of breast nodules. In our study, higher Emax values were associated with malignancies, while lower Emax values effectively predicated benign disease in breast nodules. This observation is in good agreement with the results of a previously published study^24^. There are many studies focused on SWE diagnosis, but the Emax cutoff value, sensitivity and specificity were different^25, 26^. Berg *et al*. calculated an Emax cutoff value of 80 kPa, which yielded sensitivity and specificity of 97.2% and 78.5% respectively in a multicenter study comprising 939 patients^24^. In our study, the Emax cutoff value 55.86 kPa yielded a sensitivity and specificity of 83.30% and 88.90% respectively. Even if the sensitivity was reduced in our study, the specificity was improved. The reasons for these differences might be due to the different sample size and inclusion criteria of studies.

It is well accepted that cancer cells cause a complicated stromal reaction, which results in collagen remodeling that promotes invasive behavior^27^. The stromal reaction of most benign nodules is much weaker, so they grow slowly and display no invasive behavior. The findings in this study are in agreement with these published observations^28, 29^. In addition, our ongoing study found that the stiffness of breast lesions correlates with the arrangement and shape of collagen fibers. Our results showed that breast nodules with higher Emax values had relatively higher collagen intensity than benign nodules (80.25 ± 8.51 vs. 30.77 ± 5.32, p < 0.001). Compared with malignant nodules, benign nodules were also softer and expressed much less collagen and Kindlin-2. It is thus easy to understand that elastography values may correlate with histological characteristics of a breast nodule.

Studies have reported that Kindlin-2 acts as an important regulator of integrin activation^11, 12^. Kindlin-2 is also reported to be highly expressed in various breast cancer cell lines^30^, and is known to affect cell differentiation, survival and migration^13, 14, 31, 32^. Deregulation of Kindlin-2 had been observed in various types of human cancers. For example, Gozgit *et al*. showed that Kindlin-2 promotes breast cancer cell invasion^33^ and Yoshida *et al*. found that Kindlin-2 promotes the progression of pancreatic cancer^34^. Another study also found that Kindlin-2 can up-regulate fibrosis related genes such as Col I, α-SMA, and Snail^16^. In our study, we analyzed the relationship among collagen intensity, Kindlin-2 expression, and Emax in breast nodules. We found that Kindlin-2 expression was higher in malignant breast nodules than in benign nodules (8231.77 ± 4596.01 vs. 885.75 ± 417.77, p = 0.003). Kindlin-2 expression also correlated well with Emax (coefficient = 0.88, p < 0.001). To our knowledge, this is the first study to examine the relationship between Kindlin-2 expression and breast nodule stiffness.

We demonstrated that there is a strong correlation between collagen intensity and Emax (coefficient = 0.89, p < 0.001), which is consistent with previous study^35^. Collagen is an important factor of ECM, and may affect tumor stiffness. Moreover, Kindlin-2 is a known activator of TGFβ signaling that can promote renal fibrosis^16, 36^. Based on these evidences, we boldly speculate that in breast cancer, Kindlin-2 might activate molecular signaling pathways, such as integrin signaling and/or TGFβ signaling, to induce collagen expression, re-arrangement and changes in fiber shape, and ultimately affect breast nodule elasticity.

Sentinel or axillary lymph node metastasis is very common in breast cancer patients, and effective disease management depends on availability of accurate radiological data. In our study, malignant nodules with lymph node metastasis displayed a relatively higher Emax than those that without lymph node metastasis, but it has no statistical significance (182.66 ± 89.22 kPa vs. 77.11 ± 5.84 kPa, p = 0.110). Additionally, there was no difference in collagen intensity between the metastasis group and the non-metastasis group (79.54 ± 10.22 vs. 81.35 ± 5.10, p = 0.529). We believe that a larger sample size may achieve statistical significance in this case. Kindlin-2 expression, however, was statistically higher in the nodules with lymph node metastasis than in those that without lymph node metastasis (12294.57 ± 2998.21 vs. 5502.31 ± 1669.94, p = 0.027).

Moreover, the expression of p-FAK and p-Smad2 were both higher in malignant nodules than that of benign nodules (1847.14 ± 287.91 vs. 30986.63 ± 21150.24, p = 0.020 for p-FAK expression, and 2574.87 ± 225.66 vs. 57786.39 ± 23771.11, p = 0.006 for p-Smad2 expression). Meanwhile, the expression of both p-FAK and p-Smad2 in malignant nodules with metastasis was higher than that without metastasis (50275.91 ± 1303.44 vs. 11697.36 ± 635.89, p < 0.001 for p-FAK expression, and 73890.35 ± 23007.70 vs. 41682.42 ± 10261.39, p = 0.035 for p-Smad2 expression). Therefore, these results suggested that Kindlin-2 might influence breast cancer elasticity and improve metastasis by activating integrin and TGFβ signaling pathways.

Previous studies reported that hypoxia^37^ and ECM stiffness and remodeling, fiber alignment and crosslinking can promote tumor progression and metastasis^38^. Another study reported that the collagen alignment can promote breast cancer cells migration^39^. Although, in our study, we found that Kindlin-2 might improve lymph node metastasis by activating integrin and TGFβ signaling pathways, the detailed molecular mechanisms underlying Kindlin-2 overexpression in breast cancer metastasis are unclear. Therefore, further studies focusing on the Kindlin-2 related molecular mechanisms of breast cancer progression and metastasis are needed.

We conclude on the basis of these observations that Kindlin-2 expression might be positively correlated with stiffness, degree of malignancy, and metastatic potential. Collagen plays a dynamic role in the breast cancer microenvironment, and promotes tumor progression^40^. A study in mouse models also showed that collagen fibers exert physical tension on epithelial cells and activate specific signaling pathways responsible for breast cancer invasiveness^41^. Kindlin-2 is reported to have an intimate relationship with breast cancer, as it promotes disease progression and metastasis^30^. Another study found that stiffness measured by SWE is an independent risk factor for poor prognosis in breast cancer^42^. Our results showed that Emax, a stiffness parameter, was related to collagen intensity, Kindlin-2 expression, and associated with lymph nodes metastasis. A combination of Emax, collagen content and Kindlin-2 expression might form a panel of diagnostic parameters for malignancies and associated with lymph node metastasis. Therefore, SWE is a simple, non-invasive diagnostic technique that can be useful in detection and diagnosis of breast focal diseases. In conjunction with collagen and Kindlin-2 assessment, elastography parameters can also be used as predictors of metastatic potential, and set direction for surgical decisions.

## Patients and Methods

### Patients

A prospective study was conducted in our hospital between September 2016 and December 2016. Two hundred and seventy-four patients with 302 breast nodules had undergone surgery or core needle biopsy. Patients had routinely undergone ultrasound and elastography before surgery or core needle biopsy (all of patients volunteered to undergo SWE). Exclusion criteria included nodules with near field or inner macro-calcification, since micro-calcifications may impede the SWE procedure. Other exclusion criteria were pregnancy, lactation, breast implants, ongoing radiation and chemotherapy, and presence of scars close to breast lesions. Finally, we included 102 nodules from 102 patients with an average age of 52.11 ± 10.76 years (Table 4).

**Table 4:** Characteristics of patients and breast nodules.

Parameter	Benign	Malignant
Patients (n = 102)	n = 64	n = 38
Age (y)	48.78 ± 7.87	53.78 ± 11.79
Sex	Female	Female
Size (cm)	2.18 ± 1.36	2.50 ± 0.91
Metastasis (n = 23)		23/38

Histologic analysis confirmed 64 benign nodules (43 fibroadenomas, 11 adenoses, and 10 intraductal papillomas) and 38 malignant nodules (all were invasive breast cancer). The basic information of the patients is shown in Table 1. This study was approved by our local Ethics Committee of the Chinese PLA General Hospital. Informed consent was obtained from all patients included in the study. The study was performed in accordance with relevant guidelines and regulations.

### Imaging

All the patients included in this study underwent ultrasound and SWE before surgery or core needle biopsy, so we had no prior knowledge of the pathologic results. We collected all of the SWE images and pathologic results. All SWE examinations were performed by doctor Li, a professor with more than 15 years of breast ultrasound scanning experience and at least 5 years of breast elastography experience. In brief, bilateral breast ultrasonography of every patient was performed using an ultrasound device Aixplorer (SuperSonic Imagine, Aix en Provence, France) equipped with a sonoelastography unit and a 4- to 10.0-MHz linear-array transducer. In B-mode ultrasound, the nodule echogenicity, margin, shape, and types of calcification were recorded, as well as suspicious axillary lymph nodes, which were characterised by round shape, thickened outer wall and disappearing hilus structure^43^. SWE parameters (Emax, Emin, and Emean) were obtained immediately after B-mode ultrasound using the same real-time instrument and probe. In our ultrasound device Aixplorer, blue represents softer tissue while red represents harder tissue. A colour map representing elasticity values in kilopascals (kPa) was obtained. SWE was performed using the penetration mode, with a colour scale ranging from 0 (blue) to 180 kPa (red). The SWE acquisition was performed in many planes to find stiffness areas using the SonicSoftware tool (SupersonicImagine, Aix en Provence, France). The penetration mode helps to increase the signal-to-noise ratio. A large region of interest was manually positioned so as to cover the whole lesion, including the edges. External compression was not applied and patients were asked to relax and remain still (hold their breath for a few seconds)^44^. It is well accepted that uniform blue colour in the upside region of interest indicates that there is no external compression. We ensured uniform blue colour in the upside area of interest by gently lifting the probe and adjusting pressure in areas that displayed other colouration, or by asking patient to remain still. Then we selected an area, covering the stiffest lesion. The ultrasound system automatically calculated SWE parameters (Emax, Emin, and Emean) immediately after we chose the target and reference lesions. Each lesion was assessed at least three times, and the average parameters measurements was recorded.

### Immunohistochemical (IHC) and Sirius red staining

Paraffin-embedded tissues were cut into 5-μm sections and baked at 65 °C for 30 min. Deparaffinization and hydration procedures were performed. The sections were then treated with 0.3% hydrogen peroxide for 30 min (to abolish endogenous peroxidase activity), and microwaved in 10 mM sodium citrate buffer (pH 6.0) for 20 min (for antigen retrieval). Monoclonal anti-Kindlin-2 (1:100 dilution; Abcam, USA), anti-p-FAK (1:100 dilution; Abcam, USA) and anti-p-Smad2 (1:50 dilution; Abcam, USA) antibodies were added and incubated overnight at 4 °C. Then PV60012-step plus Poly-HRP anti-rabbit IgG detection system (Zhong Shan Jin Qiao, Beijing, China) was applied and tissues incubated at room temperature for 30 min, followed by 5 min incubation with diaminobenzidine (DAB) at room temperature for colour development. Then the sections were counterstained with haematoxylin and examined under an Olympus BX51 microscope (Olympus, Tokyo, Japan)^45^. The integrated optical density of positive reactions was analyzed by using Image Pro Plus 6.0 software^46^. Additional sections were incubated for 30 minutes in 0.1% Sirius red F3B (Sigma Chemical Co.) containing saturated picric acid and 0.1% fast green^47, 48^. After rinsing twice with distilled water, the tissue sections were briefly dehydrated with 70% ethanol. Sirius red stained sections were examined under an Olympus BX51 microscope (Olympus, Tokyo, Japan), and analyzed by using the Image Pro Plus 6.0 software^46^. In each sample, three high-power fields (×200) were randomly selected, photographed, and total staining intensity was quantified.

### Statistical analysis

All statistical analyses were performed with the Statistical Package for Social Sciences (SPSS for Windows, Version 19, Chicago, IL, USA). Statistical analysis of differences in test parameters was performed with Student’s t-test or one-way analysis of variance (ANOVA). Correlations among Sirius red staining score, IHC score, and SWE elastography parameters of breast nodules were evaluated with Pearson’s test, and correlation coefficients were calculated. Coefficients ≥0.7 indicated a strong correlation; values ≥ 0.4 but <0.7 indicated a moderate correlation; and values < 0.4 indicated a weak correlation.

### Data Availability

The data generated during the current study are not publicly available as another related study is still in progress, however, these data are available from the corresponding author on reasonable request.
